# CO_2_ Adsorption by CMK-3 at Low Temperatures and High Pressure to Reduce the Greenhouse Effect

**DOI:** 10.3390/ma17153845

**Published:** 2024-08-03

**Authors:** David Cantador-Fernandez, Rocio Otero-Izquierdo, Pascal Van Der Voort, José Ramón Jiménez, José María Fernández-Rodríguez

**Affiliations:** 1 Departamento de Química Inorgánica e Ingeniería Química, E.P.S. de Belmez, Universidad de Cordoba, Avenida de la Universidad s/n, Belmez, E-14240 Cordoba, Spain; p12cafed@uco.es; 2 Instituto Químico para la Energía y el Medioambiente (IQUEMA), Universidad de Cordoba, E-14071 Cordoba, Spain; b42otizr@uco.es; 3 Centre for Ordered Materials, Organometallics and Catalysis (COMOC), Ghent University, Krijgslaan 281-S3, B-9000 Ghent, Belgium; pascal.vandervoort@ugent.be; 4 Departamento de Ingeniería Rural, E.P.S. de Belmez, Universidad de Cordoba, Avenida de la Universidad s/n, Belmez, E-14240 Cordoba, Spain

**Keywords:** CO_2_ capture, CO_2_ isotherms, CMK-3, adsorption cycles, climate change

## Abstract

In this study, the maximum CO_2_ capture capacity of an ordered mesoporous carbon (CMK-3) was evaluated at high pressure (35 atm) and several temperatures (0, 10, 20, and 35 °C). CMK-3 was synthesized with the hard template method (silica SBA-15) using furfuryl alcohol and toluene as carbon sources. The CO_2_ adsorption isotherms were fitted to the following adsorption theories: Freundlich, Langmuir, Sips, Toth, Dubinin–Radushkevich, and Temkin. The maximum capture capacity (726.7 mg·g^−1^) was achieved at 0 °C and 34 atm. The results of the study of successive adsorption–desorption cycles showed that multi-cycle reversible gas capture processes could be used in optimal temperature and pressure conditions. It was determined that 0.478 g of CMK-3 would be required to reduce the CO_2_ concentration in 1 m^3^ of air to pre-industrial levels (280 ppm). The obtained results may contribute to technological developments for the mitigation of human impacts on the environment through the capture of atmospheric CO_2_.

## 1. Introduction

Carbon dioxide (CO_2_), an essential gas for life on earth, is one of the gases responsible for the greenhouse effect (GHG). These non-polluting gases maintain the earth’s temperature in equilibrium by retaining energy from solar radiation. However, a rapid increase in the concentration of GHGs would cause an imbalance in the earth’s energy balance. The most immediate effect is a sudden increase in average global temperature, leading to changes in climate and stress in many ecosystems. Since the beginning of the 20th century, an increase of approximately 1.1 °C has been observed. This rapid increase seems to be related to anthropogenic CO_2_ emissions since the Industrial Revolution, which amount to 1.5 trillion tonnes of CO_2_, with 47% growth in the last ten years [1,2]. Most anthropogenic CO_2_ emissions are produced by the building sector and the burning of fossil fuels for use in transportation and electricity production [3].

Recent studies indicate a trend that could endanger the lives of many ecosystems and, ultimately, human life. Therefore, numerous international meetings have been held to discuss the impact of these emissions and to implement mitigation, adaptation, and support measures. One of the most prominent was the Paris Agreement (COP21) [4], which established the objective of keeping the earth’s temperature below 2 °C of pre-industrial levels.

One such measure is CO_2_ capture, which has recently gained momentum. CO_2_ capture can be performed in two main ways as follows: at the point of origin of the emissions and directly from the atmosphere. In both cases, the capture technology is defined by its destination. In carbon capture and storage CCS technology, captured CO_2_ is permanently stored, whereas carbon capture and utilisation CCU technology adds value to CO_2_ in the form of new materials or fuels.

In this context, the search for new materials for use as CO_2_ absorbers has been the focus of many scientific studies, including silica [5,6,7], hydrotalcites [8,9], periodic mesoporous organosilicas (PMOs) [10,11,12], metal–organic frameworks (MOFs) [13,14,15], porous carbons [16,17,18,19], and CMK-3, which is the subject of this study.

Porous carbons are important materials owing to their variety of applications and low costs. Activated carbon (AC) is the best-known and most widely used variant. The main advantage of this material is that it can be economically produced on a large scale through the carbonisation of plant sources. In addition, it has a high degree of porosity and a high specific surface area. The major disadvantages are its disordered structure and pore size, which is generally in the micropore range (<2 nm), leading to poor capture performance at pressures above 1 atm [20]. Therefore, it is necessary to study the use of mesoporous carbon in CO_2_ adsorption at high pressures.

Pino et al., 2016 [17] and Wang et al., 2013 [18] used commercial carbon black and mesoporous carbon, respectively, both modified with amines (PEI). The results of their studies were obtained at atmospheric pressure. Casco et al., 2014 [19] used carbon from an aliphatic petroleum residue modified with KOH as the activating agent, and Sevilla et al., 2018 [21] used carbon modified with potassium oxalate. The experiments were performed at 45 and 50 atm, respectively, and at 25 °C. Adsorption and desorption cycles were not performed to determine the stability of the material.

In 1999, Ryoo et al. [22] first described the synthesis of a highly ordered mesoporous carbon belonging to a group of novel materials called mesostructured carbon by KAIST (CMK-n). First-order mesoporous carbon, CMK-1, was synthesised from the template silica MCM-48 by Kruk et al. in 2000 [23]. Later, in 2000, Jun et al. [24] synthesised the first hexagonally ordered mesoporous carbon, CMK-3, from template silica SBA-15. Other studies used different carbon sources, such as glucose, xylose, sucrose, and acenaphthene.

**Figure 1:**
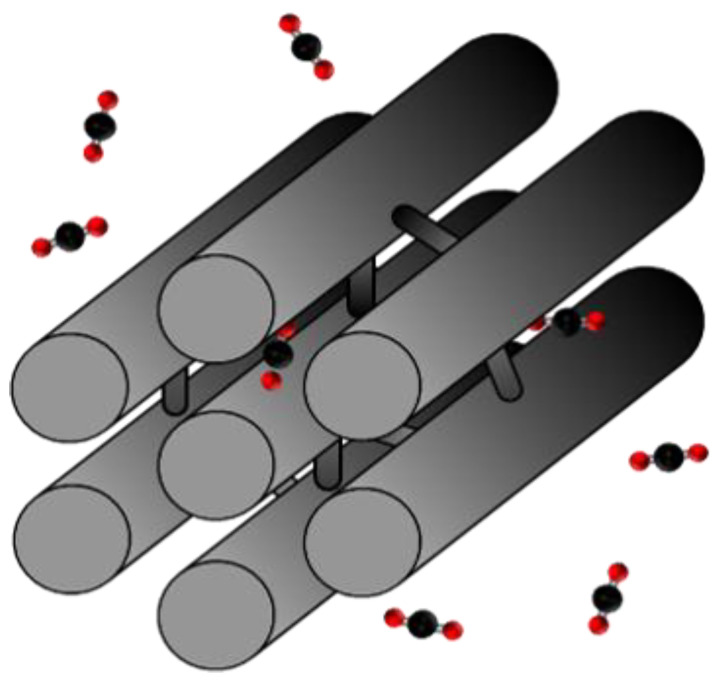
Representation of the CMK-3 structure.

The results of these studies show a material (CMK-3) composed of carbon nanorods arranged in space, forming a hexagonally ordered porous network in the small mesopore range (Figure 1). Its porous structure and high specific surface area give it a high adsorption potential. Additionally, the ease of synthesis, together with the low production cost, makes its scalability feasible at the industrial and commercial levels.

This characteristic makes CO_2_ adsorption favourable at both high and low pressures. In addition, mesopores facilitate the rapid passage of adsorbates into smaller pores [25]. These properties, together with a high specific surface area and large pore volume, are key to obtaining the best adsorption performance for gases such as CO_2_.

Several studies have been conducted on CO_2_ adsorption using CMK-3. Among them, studies at low pressures [26] and high pressures [27,28,29] did not include analyses of adsorption behaviour at different temperatures and adsorption loss from successive adsorption–desorption cycles. These analyses are essential for its commercial application, as they allow for the determination of the degree of reversibility and reusability of the adsorbent material.

Su et al., 2018 [29] modified CMK-3 with MDEA synthesised from sucrose (the present study used furfuryl alcohol and toluene as carbon sources [30]) and studied its adsorption in the presence of water.

In this study, ordered mesoporous carbon CMK-3 was selected as CO_2_ adsorbent. The capture capacity was determined at high pressures (34 atm) and different temperatures (0, 10, 20, and 35 °C), whose isotherms were analysed according to various adsorption theories to determine the adsorbent–adsorbate behaviour. Finally, a capture capacity study was conducted after 10 successive adsorption–desorption cycles.

Under optimal temperature and pressure conditions, this material can be used in multicyclic processes of reversible gas capture for subsequent applications in reuse or storage technologies. Some applications of this adsorbent include its introduction into cement-based materials. The results of this study will contribute to technological developments for the mitigation of human impacts on the environment.

## 2. Materials and Methods

### 2.1. CMK-3

Ordered mesoporous carbon (CMK-3) was synthesised to obtain a base carbon material with ordered mesoporosity and a high specific surface area (Figure 1). The hard template method proposed by Niebrzydowska et al., 2013 [30] was used for this purpose.

First, 2.5 g of template SBA-15 (Sigma-Aldrich, St. Louis, MO, USA) was impregnated with a 10 mL solution of 25 vol % furfuryl alcohol in toluene (Sigma-Aldrich) as the carbon source. To create a favourable acidic medium, 200 μL of H_2_SO_4_ (0.1 mol·L^−1^) was added. The solution was refluxed and stirred for 36 h at 90 °C. The silica was washed with toluene three times and then oven-dried (150 °C for 5 h). The resulting material was carbonised for 1 h at 1100 °C in an inert atmosphere (Ar-5% H_2_). Finally, to remove silica from the template, stirred leaching was performed for 6 h using 10% hydrofluoric acid (HF) (Sigma-Aldrich).

### 2.2. Material Characterisation

The characterisation of mesoporous carbon CMK-3 was performed using previously described techniques [9,12]. X-ray diffraction (XRD) was performed using the Bruker D8 Discover A25. The scanning rate was 0.02° (2θ)·s^−1^ between 3° (2θ) and 70° (2θ). The International Database ICDD 2003 [31] was used to identify the crystal structure. The particle size was measured by laser diffraction using a Mastersizer S instrument (Malvern Instruments, Malvern, UK). To disaggregate the material particles, an ultrasonic bath was used for 10 min before measurement, and ethanol was used as a dispersant. N_2_ adsorption isotherms were obtained using an Autosorb iQ2 instrument (Quantachrome Instruments, Boynton Beac, FL, USA). The sample was subjected to vacuum (5 × 10^−10^ mbar) at a temperature of 70 °C 24 h before the measurement. The N_2_ adsorption–desorption isotherm was performed at −196 °C between 0 and 1 atm. AsiQwin 3.0 software (Quantachrome Instruments) was used to analyse the results. The Brunauer–Emmett–Teller (BET), single-point, and Barrett–Joyner–Halenda (BJH) methods were used to calculate the BET surface areas, pore volumes, and pore size distributions, respectively. A Talos F200i ThermoFisher Scientific (Waltham, MA, USA) was used for transmission electron microscopy. The CO_2_ isotherms and adsorption–desorption cycles were obtained using a Sievert PCTPro-2000 (Setaram, Caluire, France) with an auxiliary Julabo F250 refrigeration unit. To achieve greater precision, a MicroDoser accessory with 0.5 mL of sample holder volume was used. The equilibrium adsorption was set at 0.1 [wt. %·min^−1^] × 1000 rate limit. Two hours before the measurement, the sample was subjected to high vacuum (1 × 10^−7^ hPa). The gas purities were CO_2_ (99.995%) and He (99.999%).

### 2.3. Adsorption Isotherms

To describe the adsorption processes occurring on the surface of the CMK-3 adsorbent, the CO_2_ adsorption curves were fitted using the following six adsorption theories: Langmuir [32,33], Freundlich [34], Sips [35], Toth [36], Dubinin–Radushkevich (D-R) [37,38], and Temkin [39,40]. Calculations were performed using MATLAB-R2015a software. Adsorption theories commonly found in the literature include the Langmuir, Freundlich, Dubinin–Radushkevich, and Temkin models. These two-parameter models describe simple adsorption curves in which only monolayer filling occurs. When we need to analyse more complex curves with one or several concavity changes, where a change in the adsorption process, such as multilayer filling, has occurred, these models cannot describe the adsorption well [41]. Therefore, we must use models with at least three parameters, which are the Sips and Toth models. These models combine the maximum capture capacity or plateau parameters of the Langmuir model and the adsorbent–adsorbate interactions of the Freundlich model. Cantador et al., 2021 [9] discussed these models in detail.

The Langmuir model (1918) describes the formation of a monolayer on a homogeneous surface. There is an adsorption limit, and no allowance is made for variations in the adsorption energy (Equation (1)):

(1) $$q=\frac{q_{m}\cdot K_{L}\cdot Pr}{1+K_{L}\cdot Pr}$$

where *q* is the adsorption capacity (mg·g^−1^), *Pr* is the relative pressure (p·p_0_^−1^), *q_m_* is the monolayer capacity (mg·g^−1^), and *K_L_* is the Langmuir equilibrium constant (atm^−1^).

This model was rectified by Toth by introducing a correction factor X_L_ [9,36] to improve the prediction of the maximum capacity of the monolayer (*q_mc_* instead of *q_m_*). As this model describes adsorption in a monolayer over its entire surface area, it is possible to calculate the specific surface area (*S_L_*) (Equation (2)):

(2) $$S_{L}=\left(\frac{q_{mc}\cdot 22414}{1000\cdot 44.01}\right)\cdot N\cdot \frac{A}{22414}$$

where *S_L_* is Langmuir surface area (m^2^·g^−1^), *q_mc_* is the monolayer capacity corrected by Toth (mg·g^−1^), N (mol^−1^) is the Avogadro’s number, and *A* is the cross-sectional area of the adsorbate (0.17 nm^2^ for CO_2_).

Freundlich’s model (1926) describes monolayer formation on heterogeneous surfaces and allows for a better fit of less intense or linear curvatures at low pressures. There is no adsorption limit, which allows for the description of isotherms in which the adsorption energy is dependent on the adsorbed quantity (Equation (3)):

(3) $$q=K_{f}\cdot {Pr}^{\frac{1}{nf}}$$

where *K_f_* ((mg·g^−1^)·(atm^−1/n^)) is the Freundlich constant and *n* (1/*nf*) is the heterogeneity factor.

The Sips model unifies the two aforementioned models. It considers adsorbent–adsorbate interactions with varying adsorption energies and describes the maximum adsorption capacities at high pressures using the Langmuir model. Similar to the Langmuir equation, Toth introduced the correction factor *χ_S_*. The combination with the C model allows for transformation into a multi-layer fitting model (Equation (4)):

(4) $$q=\frac{q_{S}\cdot \chi_{S}\cdot {Pr}^{ns}}{\left(\frac{1}{K_{S}}+{Pr}^{ns}\right)\cdot (1-k\cdot ∆Pr)}$$

where *q_S_* is the maximum adsorption capacity (mg·g^−1^), *K_S_* is the Sips equilibrium constant (atm^−1^), *χ_S_* is the thermodynamic correction factor by Toth, *ns* the heterogeneity factor, *k* is a factor related to the number of adsorption layers, and Δ*Pr* is a parameter that indicates the point at which the formation of multilayers begins.

The Toth model (1971) modifies the Langmuir equation, allowing for a good fit to adsorption isotherms with monolayer and multilayer formations. A new constant *a_T_* relative to the binding affinity was introduced (Equation (5)):

(5) $$q=\frac{q_{T}\cdot a_{T}\cdot Pr}{{\left(\frac{1}{K_{T}}+{Pr}^{nT}\right)}^{1/nT}\cdot (1-k\cdot ∆Pr)}$$

where *q_T_* is the maximum adsorption capacity (mg·g^−1^), *a_T_* is a constant related to the binding affinity (*a_T_* = (1 + 1/*K_T_*)^1/*nT*^), *K_T_* is the Toth equilibrium constant (atm^−1^), and *n_T_* the heterogeneity factor.

The Dubinin–Radushkevich model (1935), based on Polanyi’s theory, relates the adsorbent–adsorbate potential energy (*ε*) at pore filling to the adsorption capacity (Equation (6)):

(6) $$q=q_{D}\cdot e^{\left(-\beta \cdot \varepsilon^{2}\right)}$$

where *q_D_* is the volumetric filling capacity of the micropores (mg·g^−1^), *β* (mol^2^·kJ^−2^) is the affinity coefficient or lateral interaction energy, and ε the potential energy between the adsorbent and the adsorbate (kJ·mol^−1^).

This model, based on the affinity coefficient, makes it possible to calculate the free energy of adsorption and identify the nature of adsorption.

The Temkin (1940) model relates the amount of gas adsorbed to the heat generated during adsorption. The energy is considered to be uniform over the entire surface area (Equation (7)):

(7) $$q=B\cdot \ln\left(K_{Tk}\cdot P\right)$$

where *B* = *RT*/*b_Tk_*, *b_Tk_* is the Temkin constant related to the variation in adsorption energy (kJ·mol^−1^), and *K_Tk_* is a constant corresponding to the maximum binding energy (atm^−1^).

## 3. Results and Discussion

### 3.1. Characterisation

**Figure 2:**
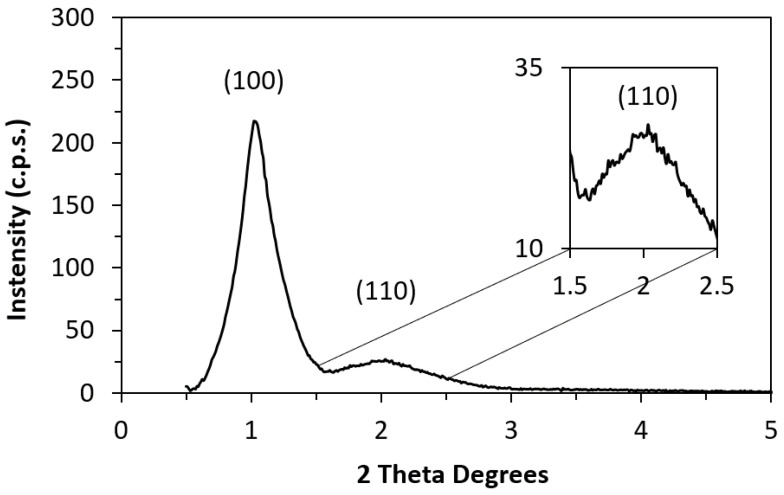
XRD pattern of CMK-3.

Structural and textural characterisations of ordered mesoporous carbon (CMK-3) were performed. Figure 2 shows a diffractogram corresponding to the X-ray diffraction (XRD) test. Two peaks were detected as follows: a main peak (100) at d = 85.7 Å and a secondary peak (110) at d = 43.9 Å, which are in agreement with previous results obtained for the same material [20] and correspond to a 2D hexagonal symmetrical structure.

**Figure 3:**
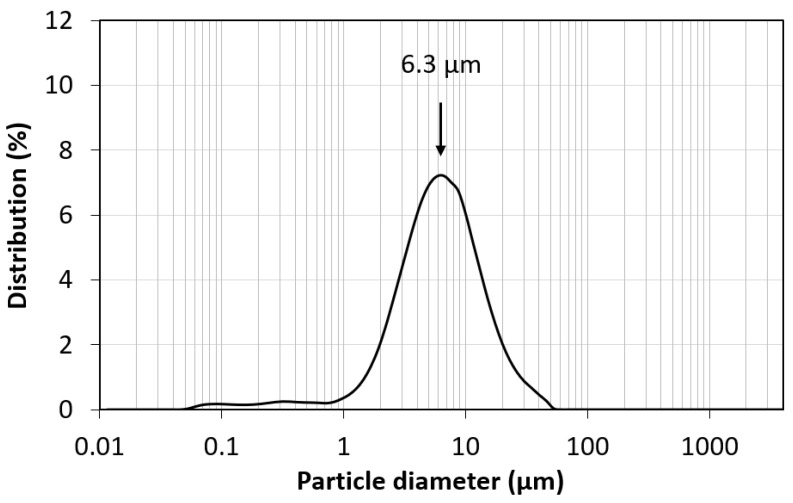
Particle size distribution curve for CMK-3.

Figure 3, corresponding to the particle size test, shows an unimodal type distribution curve located between 0.8 μm and 14 μm and with a peak centred at 6.3 μm, indicating that the size lies in a narrow and very similar range for all the particles in the sample.

**Figure 4:**
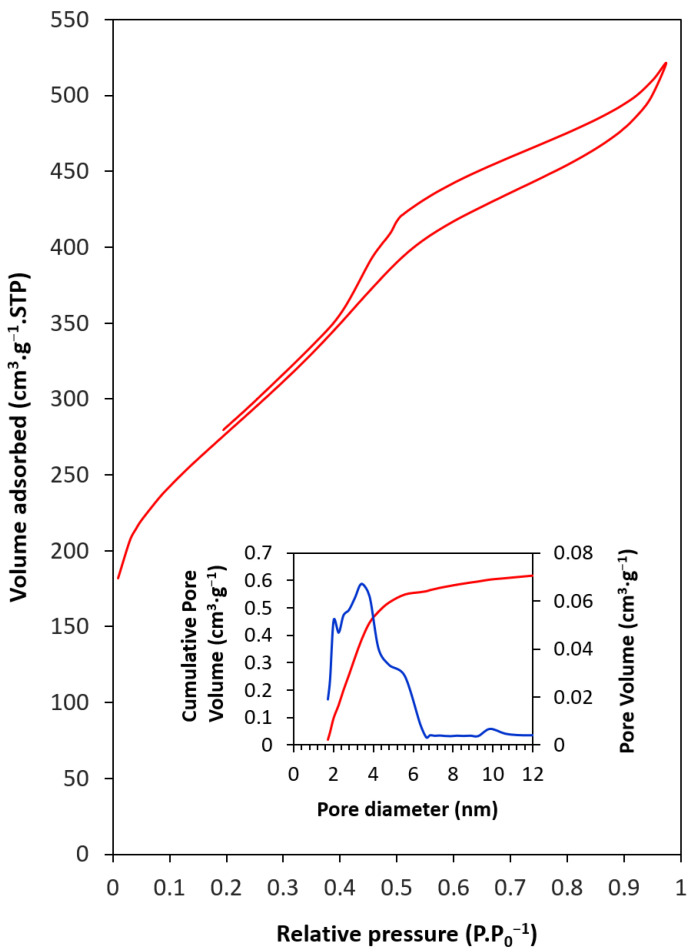
Nitrogen adsorption–desorption isotherm and pore size distribution of CMK-3. In the inserted figure: (Blue line) Pore volume, (Red line) Cumulative pore volume.

Adsorption–desorption analysis with N_2_ was performed (Figure 4). The results showed a Type IVa adsorption curve (IUPAC). This curve is associated with monolayer–multilayer adsorptions in mesoporous materials, and at relative pressures close to 1, the curve forms a plateau (p·p_0_^−1^ ≈ 0.53 atm), which is related to capillary condensation. The adsorption curve also shows Type H1 hysteresis, which is associated with a narrow range of uniform cylindrical mesopores. The BET method results showed a high specific surface area of 990 m^2^·g^−1^, of which 198 m^2^·g^−1^ correspond to micropores (20% of the total). The mesoporosity of the material corresponds to the space between the carbon nanorods of the hexagonal lattice, whereas the microporosity is related to the porosity of the carbonaceous material itself [26]. The BJH method was used to calculate the volume and pore size distribution. The pore size range was 1.7 nm or less (micropores) and 6.7 nm in diameter (small mesopores). The pore volume obtained was 0.77 cm^3^·g^−1^ (Table 1).

**Figure 5:**
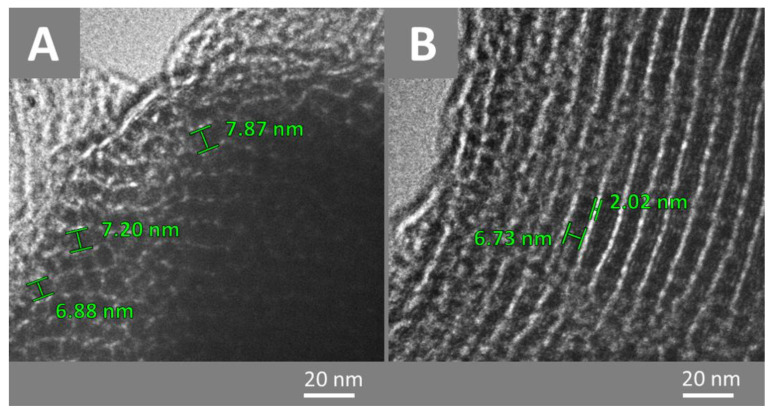
TEM images of CMK-3. (**A**) vertical view, (**B**) horizontal view. In black, nanorods; in white, channels.

High-resolution transmission electron microscopy (HTEM) was performed to corroborate the pore size obtained by adsorption–desorption analysis with N_2_. Figure 5 shows two CMK-3 particles with the hexagonal arrangement confirmed by the X-ray diffraction. This arrangement revealed a set of nanorods with diameters ranging from 6.8 to 7.8 nm. Between these nanorods would be channels parallel to the nanorods, with diameters greater than 2 nm, which could be referred to as small mesopores.

### 3.2. CO_2_ Adsorption

**Figure 6:**
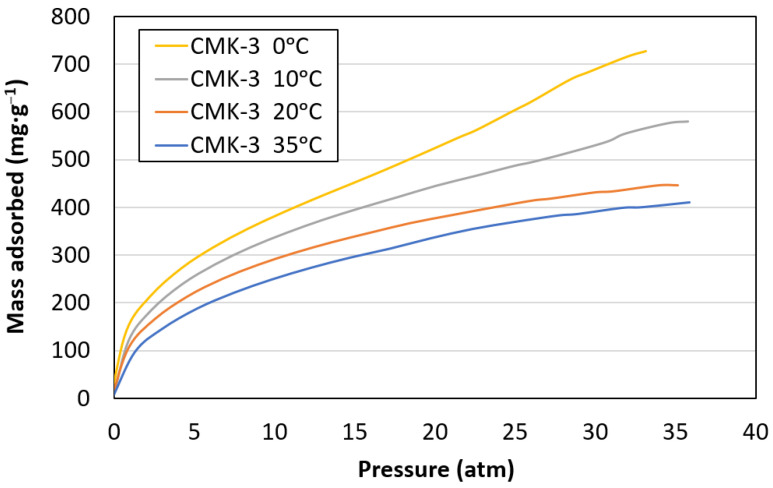
CO_2_ adsorption equilibrium isotherms of CMK-3 at 0 °C, 10 °C, 20 °C, and 35 °C.

Figure 6 shows the CO_2_ adsorption curves for CMK-3 at 0, 10, 20, and 35 °C between 0 and 35 atm. The curves for 35 °C and 20 °C showed a monolayer adsorption behaviour up to 35 atm, whose maximum adsorption capacity had not been reached. For the samples tested at 0 °C and 10 °C, the end of monolayer filling and the beginning of multilayer formation were observed at approximately 20 atm (relative pressure p·p_0_^−1^ = 0.57) and 23.7 atm (relative pressure p·p_0_^−1^ = 0.68), respectively. The filling of the multilayer led to an increased adsorption capacity. An increase in the amount of CO_2_ adsorbed was observed at decreasing temperatures, obtaining the maximum adsorption capacity at 0 °C and relative pressure p·p_0_^−1^ = 1 (726.7 m^2^·g^−1^). These results are in agreement with those obtained in previous works [8,9,12].

**Figure 7:**
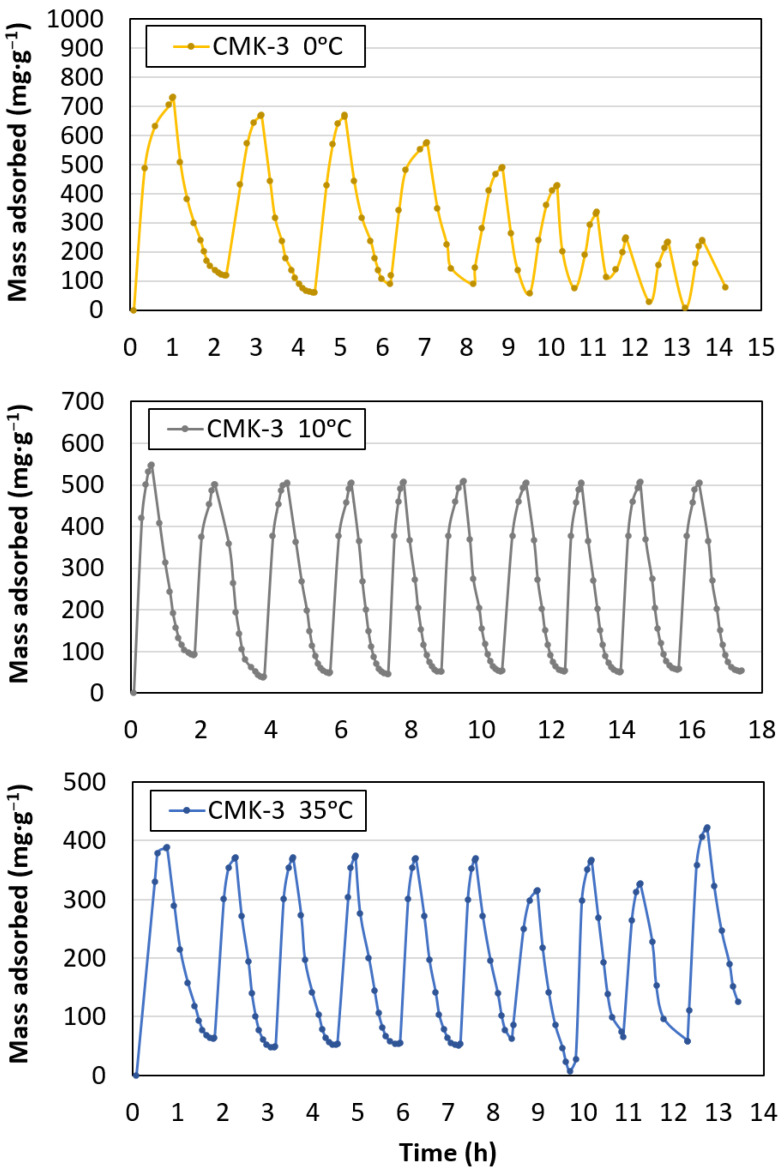
CO_2_ adsorption–desorption cycles of CMK-3 at 0 °C, 10 °C, and 35 °C.

**Figure 8:**
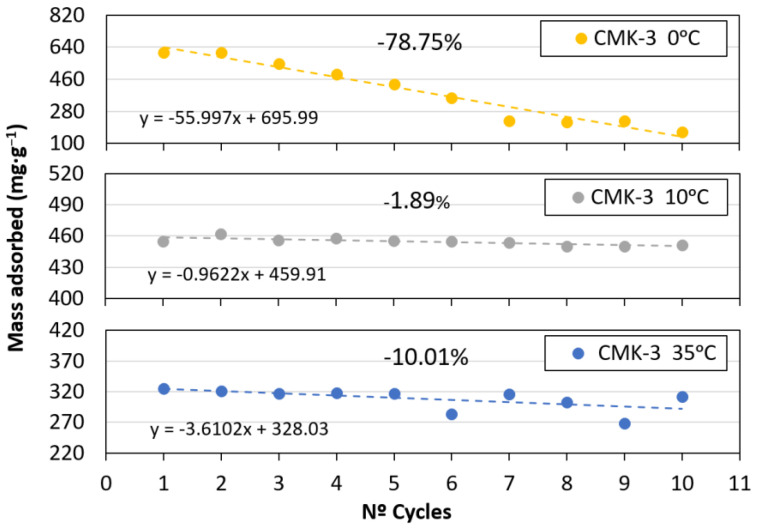
CO_2_ adsorption capacity after 10 cycles of CMK-3 at 0 °C, 10 °C, and 35 °C.

Ten adsorption and desorption cycles were performed at 0, 10, and 35 °C (Figure 7) to analyse the reversibility of CO_2_ capture and the stability of the material with respect to temperature. Figure 8 shows the maximum adsorption capacity for each cycle. A linear trend was calculated to determine the loss of capture capacity. The best performance was obtained for the CMK-3 sample tested at 10 °C, with a small capacity loss after 10 cycles (approximately 2%). A large drop in capture performance was observed after 10 cycles for the 0 °C sample. This could be due to the significant presence of micropores and small mesopores, which were very close to micropore size. Temperature is a determining factor in gas diffusion through pores. At lower temperatures, capillary and vapour condensation above the critical temperature, coupled with low diffusion and critical pore size, could be responsible for slower adsorption and partial desorption because of pore blockage. With increasing temperature, diffusion is favoured and condensation does not usually occur; therefore, adsorption is faster and desorption is complete [25,42]. Comparing the results of adsorption and loss of capture capacity with those of previous studies on CO_2_ scavengers with a low number of micropores, such as ethane–PMO [12] (whose proportion represents only 0.25% of the total pores), showed that this phenomenon does not occur. Furthermore, comparing the shapes of the adsorption curves for both studies confirmed that microporosity is the main factor responsible for adsorption at low pressures [26], as it occurs in CMK-3. The sample at 35 °C exhibited low adsorption stability from cycle number five onwards. The results revealed that the CMK-3 adsorbent could be reused for at least 10 cycles without appreciable loss of its CO_2_ adsorption properties.

**Figure 9:**
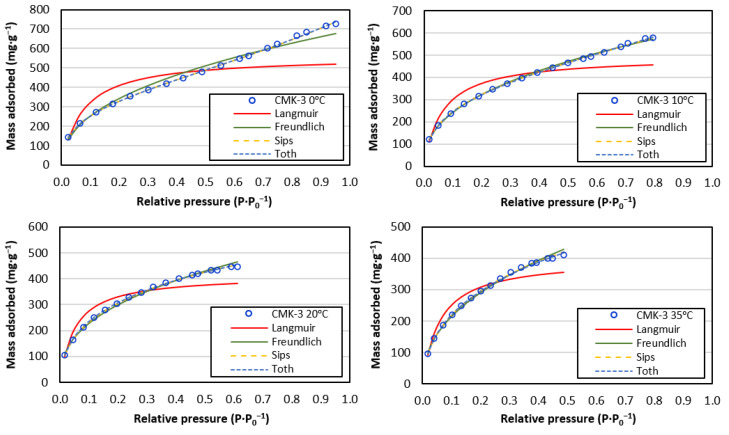
Fit curves of Langmuir, Freundlich, Sips, and Toth models for CMK-3.

To describe the adsorption processes, the CO_2_ adsorption curves were fitted using the Langmuir, Freundlich, Sips, Toth, Dubinin–Radushkevich, and Temkin adsorption theories (Figure 9).

All isotherms were well-fitted to the Freundlich equation; however, for the Langmuir equation, the R^2^ value was below 0.96. This was mainly due to multilayer formation and adsorption at high pressures, which did not form a well-defined horizontal plateau. Sips and Toth had the best fit (R^2^ = 1) for all temperatures because three parameters are used in their equations.

The Freundlich model describes the heterogeneity in the adsorption surface (n), which is related to the intensity of the adsorbate–adsorbate interactions (nf) and, in turn, to adsorption at low pressures. In all cases, the n values show a somewhat heterogeneous surface, and the nf values are above 1 and close to 2, implying a weak but favourable interaction [36]. According to the classification by Giles et al. (1960) [43], the shape of the isotherms agrees with a curve closer to the C-type, in which the number of adsorption sites remains constant at all concentrations up to saturation. This curve is related to the Freundlich model. The curvature of the isotherm was slightly greater and the adsorption was higher at low pressures than that observed for materials such as ethane–PMO [12]. In addition, the adsorbent–adsorbate intensity (nf) was slightly higher than that of ethane–PMO (Table 2). This is related to the larger number of micropores responsible for adsorption at low pressures.

A positive trend was observed at lower temperatures for the adsorption values per unit concentration (*K_f_*) in the Freundlich model, which was related to higher adsorption capacity at lower temperatures.

The Langmuir specific surface area (*S_L_*) of the adsorbent material was obtained by applying the Langmuir equation corrected by Toth [9,12]. At 0 °C, where the maximum relative pressure (p·p_0_^−1^ = 1) was achieved, the *S_L_* was 1215.7 m^2^·g^−1^, which is somewhat above the specific surface area for N_2_ (Table 1); this could be related to a low R^2^.

The values discussed for the Freundlich model and those related to the capture capacity for the Langmuir model (*q_mc_* and *K_L_*) adequately reproduced the evolution of the experimental isotherms with respect to temperature. This is in agreement with the literature on CO_2_ capture [8,9,12].

The Sips and Toth models improved the fit with respect to the two-parameter models, as indicated by the R^2^ values in Table 3. This is due to a higher complexity in the curves at lower temperatures (0 °C and 10 °C) where the adsorption multilayer was formed. Calculation of the pre-parameter (multilayer onset) was performed using MATLAB R2015a based on the inflection points of the curves.

For the isotherms at 0 and 10 °C, the onset of the multilayer occurred at p·p_0_^−1^ = 0.57 and 0.68, respectively. The pre-parameter of the curves with complete adsorption in the monolayer is the maximum relative pressure of the isotherm. The *qs* and *q_T_* parameters confirm the adsorption capacity trends in the previous models. The *n_S_* and *n_T_* (heterogeneity factors) agreed with the degree of heterogeneity obtained in the Freundlich model. This study highlights the value of using three-parameter models together with classical two-parameter models for the adequate evaluation of adsorption in curves with two different adsorption processes (monolayer and multilayer).

The Dubinin–Radushkevich model showed a similar fit to the Langmuir model, with R^2^ values above 0.9 at all temperatures except for 0 °C. The adsorption capacity exhibited the same trend with respect to temperature, although at slightly higher values. The free energy (E) was always less than 8 kJ·mol^−1^, indicating that it is an adsorption of physical nature. An increase in the free energy (E) with increasing temperature was observed (Table 4). The same trend was observed for the Freundlich parameter, *nf* (adsorption intensity) [9].

The Temkin model obtained R^2^ values similar to those of the Dubinin–Radushkevich model. The results for the Temkin constant (*b_Tk_*) agreed with those obtained for the Freundlich adsorption intensity (*nf*), showing a trend with respect to similar temperatures and low adsorption energies [44]. The weak intermolecular interactions between the gas adsorbent and the adsorption of the physical nature are in agreement with the reversible capture phenomena (Figure 7 and Figure 8).

Table 5 presents a bibliographical review of the most important recent publications on CO_2_ capture using different types of mesoporous materials [45]. In most of the studies presented in Table 5, the capture study was performed at a single pressure and temperature, and adsorption–desorption was not performed for several cycles. In contrast, the mesoporous material CMK-3 was subjected to a CO_2_ capture test under different working conditions, as well as adsorption–desorption and analysis of the isotherms by several adsorption theories. These analyses allowed for the study of the gas physisorption behaviour of this material for its application in capture technologies.

Suescum-Morales et al., 2021 [8] and Cantador Fernandez et al., 2022 [9] studied two types of hydrotalcites. For both cases, the capture results by CMK-3 at 0 °C and 34 atm were 5.1 and 4.1 times higher, respectively. This may be due to the higher specific surface area of CMK-3.

Some studies on periodic mesoporous organosilica (PMOs) have also been reported. Kirren et al., 2022 [46], Wei et al., 2016 [10], Liu et al., 2016 [47], and Sim et al., 2015 [48] studied the standard pressure conditions for PMO–ethane-modified, PMO–benzene-modified, and PMO-UDF. Lourenço et al., 2016 [11] worked under 10 atm of pressure. The adsorption results for CMK-3 were superior to all of these cases, working under higher pressure conditions (34 atm) and temperatures between 0 °C and 35 °C. Finally, CMK-3 was compared with another study by the same author on PMO–ethane [12]. The results for 34 atm and 0 °C showed that the CMK-3 material obtained a somewhat lower value (727 mg·g^−1^) than that of PMO–ethane (827 mg·g^−1^). However, at 35 °C, the CO_2_ adsorption was much higher for CMK-3 (404 mg·g^−1^) than that of PMO–ethane (273.9 mg·g^−1^), which indicates its higher capture capacity at higher temperatures and more constant capture capacity against temperature variation.

Moreover, the results of this study were compared with those of some mesoporous metal–organic frameworks (MOFs) obtained in the studies by Bourrelly et al., 2005 [49], Zhang et al., 2011 [50], Zhou et al., 2016 [51], Millward et al., 2005 [52], and Furukawa et al., 2010 [53]. Except for the capture results for MIL-101 (1007.6 mg·g^−1^ at 25 °C and 30 atm) [50], MOF-177 (1493 mg·g^−1^ at 25 °C and 42 atm) [52], and MOF-200 and MOF-210 (2400 mg·g^−1^ at 25 °C and 50 atm) [53], the maximum capture values for CMK-3 were higher at 0 °C and 34 atm (727 mg·g^−1^). The two best trapping results for the MOFs were obtained at working pressures higher than those used in this study. It should be noted that MOFs are unstable materials whose capture capacity can be reduced after several adsorption cycles or with an increase in temperature because of the loss of the adsorbent material [54] and sintering processes [55,56]. None of the cited studies performed adsorption–desorption cycles tests, which are of great importance for establishing the optimal conditions for capture and purification technologies for atmospheres with high CO_2_ content [57].

Mesoporous silica is another material recently used in CO_2_ capture. Sanz-Pérez et al., 2016 [5] studied SBA-15, which was used in this study as a template for CMK-3 synthesis. Sim et al., 2015 [48] studied SBA-15 silica functionalised with polyethylenimine (PEI). Niu et al., 2016 [58] and Li et al., 2015 [59] analysed PEI-functionalised nanosilica. The best capture result for these studies was 138 mg·g^−1^ at 75 °C and 1 atm. This value is lower than the values obtained in this study for all temperatures at 34 atm.

In this study, a mesoporous carbon-based material was studied, and the CO_2_ capture results were compared with those of other studies that used activated carbon as an adsorbent. Pino et al., 2016 [17] and Wang et al., 2013 [18] used commercial carbon black and mesoporous carbon, respectively, both modified with amines (PEI). The results of their studies at atmospheric pressure showed a lower performance than any of the results of the present study at 34 atm. Casco et al., 2014 [19] used carbon from an aliphatic petroleum residue modified with KOH as the activating agent, and Sevilla et al., 2018 [21] used carbon modified with potassium oxalate. The best adsorption results were 1388 mg·g^−1^ and 2160 mg·g^−1^ at 45 and 50 atm, respectively, at 25 °C. Although these values are greater than those in the present study, the pressures reached were also higher, and adsorption and desorption cycles were not performed to determine the stability of the material. In addition, the syntheses of these materials are more complex than those of CMK-3, and in the case of Casco et al., 2014 [19] two heat treatment processes were required. Zhu et al., 2015 [60] and Kayal et al., 2018 [54] modified typical adsorbents (hydrotalcite and MOF, respectively) by incorporating activated carbon. Only the modified MOF [54] obtained adsorption values higher than those of CMK-3, although, as reported, it is an adsorbent with temperature stability problems.

Finally, the results obtained were compared with other studies on CO_2_ adsorption on CMK-3 and modified CMK-3 [27,28,29]. Su et al., 2018 [29] modified CMK-3 with MDEA synthesised from sucrose (the present study used furfuryl alcohol and toluene as the carbon sources [30]) and studied its adsorption in the presence of water. The present study obtained similar adsorption results for the sample without water at 0 °C and 34 atm. Similarly, this study was extended to adsorption at different temperatures, analysing the stability of the material over 10 adsorption–desorption cycles.

The amount of the CMK-3 adsorbent required to reduce the current average atmospheric concentration of CO_2_ (421 ppm) [61] to the pre-industrial level (280 ppm) [62] was calculated. Aiming at future industrial use of this adsorbent material, considering the best capture performance after 10 adsorption cycles (579 mg·g^−1^ at 10 °C) and following the same methodology as previous studies [9,12], it was determined that 0.478 g of CMK-3 would be necessary to change the atmospheric CO_2_ concentration of 1 m^3^ of air to pre-industrial levels. To reduce the atmospheric CO_2_ concentration in the great pyramid of Keops at Giza (Egypt), with a height of 138.5 m and a volume of approximately 2.6 million m^3^ [63], to pre-industrial levels, 1243.2 kg of CMK-3 would be required.

**Table 5:** CO_2_ maximum capture comparative for mesoporous materials.

Adsorbent	Tª Isotherm (°C)	Pressure (atm)	Capacity Adsorption (mg·g^−1^)	Ref.
Layered double hydroxide (LDH)				
Hydrotalcite MgAl-CO_3_^1^	0	≈35	142.02	[8]
Organohydrotalcite TDD ^1^	0	35	176.66	[9]
Periodic mesoporous organosilica (PMO)				
PMO–ethane Np ^2^ iPrbipy ^3^	0 and 25	1	99.44 and 45.7	[46]
PMO–ethane nanocubes	0	1	62.48	[10]
PMO-UDF ^4^	0	≈1	52.8	[47]
PMO–benzene	25	1	22	[48]
PMO–benzene-modified ^5^	25	1	133.32	
NH2–phenylene–PMO	25	≈10	114.4	[11]
PMO–ethane	0 and 35	≈34	827.8 and 273.9	[12]
Mesoporous metal–organic framework (MOF)				
MIL-53 (Al)	31	29	462	[49]
MIL-47 (V)	31	20	506	
MIL-101	25	30	1007.6	[50]
MIL-101(Cr,Mg)	25	1	145.2	[51]
MOF-2	25	42	143	[52]
MOF-74	25	42	457	
MOF-177	25	42	1493	
MOF-200 and MOF-210	25	50	2400	[53]
Mesoporous silica (MS)				
SBA-15	45	1	21.2	[5]
PEI-SBA-15	25	1	81.4	[48]
PEI50-MS nanotube	85	0.6	121	[58]
PEI50–nano silica	75	1	138.16	[59]
Activated carbon (AC)				
Commercial carbon black	28	1	13.2	[17]
PEI-AC	28	1	112.2	
Modified AC (VR5-4:1)	25	45	1388	[19]
Modified AC (G-3.6-2)	25	50	2160	[21]
AC–hydrotalcite	200	3	72	[60]
AC-MIL-101(Cr) MOF	27	9	1080	[54]
Ordered mesoporous carbon				
CMK-3	0	≈34	682	[29]
CMK-3-MDEA	0	≈34	550	
CMK-3	0	≈34	727	This work
	10	≈34	571	
	20	≈34	447	
	35	≈34	404	

^1^ Previous heat treatment at 500 °C for 2 h, ^2^ nanoparticles, ^3^ co-condensed method with trialkoxysilylated iPrbipyridine, ^4^ urea-derived framework, ^5^ N-[3-(trimethoxysilyl)propyl]ethylenediamine-modified.

## 4. Conclusions

In this study, the maximum capture capacity of ordered mesoporous carbon (CMK-3) and its behaviour at different temperatures and pressure up to 34 atm were determined by performing several successive adsorption–desorption cycles. The main conclusions are summarized as follows:

1. The laser particle size test showed a unimodal-type distribution curve located between 0.8 μm and 14 μm and a peak centred at 6.3 μm.
2. The pore size range was 1.7 nm or less (micropores) and 6.7 nm in diameter (small mesopores). The pore volume was 0.77 cm^3^·g^−1^. The S_BET_ was 990 m^2^·g^−1^, of which 198 m^2^·g^−1^ corresponded to micropores (20%).
3. All isotherms showed a good fit to the Freundlich model, and the Langmuir model obtained a worse fit. The obtained Freundlich values indicated a somewhat heterogeneous surface and a weak but favourable adsorbate–adsorbate interaction.
4. The Sips and Toth models improved the Langmuir and Freundlich fit, especially for low temperatures (0 °C and 10 °C) where multilayers were formed.
5. The free energy (E < 8 kJ·mol^−1^) obtained from the Dubinin–Radushkevich model was in agreement with the physical nature of adsorption. Temkin’s constant indicated a weak intermolecular adsorbent–adsorbate interaction.
6. The highest capture capacity (726.7 mg·g^−1^) was obtained for working conditions of the minimum temperature studied (0 °C) and maximum pressure (34 atm).
7. The best performance, in terms of capture capacity loss after 10 adsorption–desorption cycles, was achieved at 10 °C and 34 atm, with only a 2% loss.
8. The results showed that 0.478 g of CMK-3 would be enough to reduce the CO_2_ concentration in 1 m^3^ of air to pre-industrial levels. Consequently, 1243.2 kg of CMK-3 would be required to reduce the CO_2_ concentration within the Great Pyramid of Keops at Giza (Egypt) to pre-industrial levels.

Under optimal temperature and pressure conditions, this material is suitable for multicyclic reversible gas-capture processes. The findings of this study contribute to the development of the technologies for the capture and use of greenhouse gases such as CO_2_.

## Figures and Tables

**Table 1:** Pore structure parameters for CMK-3.

	S_BET_ (m^2^·g^−1^)	Smc ^a^ (m^2^·g^−1^)	Vp ^b^ (cm^3^·g^−1^)	Dp ^c^ (nm)
CMK-3	990	198	0.77	3.41

^a^ Micropore surface, ^b^ single-point pore volume, ^c^ diameter pore average size, calculated by BJH method.

**Table 2:** Langmuir and Freundlich model parameters for CO_2_ adsorption on CMK-3.

	Langmuir						Freundlich			
	*q_m_*	*X_L_*	*q_mc_*	*K_L_*	*S_L_*	R^2^	*K_f_*	*n*	*nf*	R^2^
	(mg·g^−1^)		(mg·g^−1^)	(atm^−1^)	(m^2^·g^−1^)		(mg·g^−1^·atm^(−1/n)^)		(1/n)	
CMK-3 0 °C	560.87	1.073	522.61	13.66	1215.7	0.888	690.93	0.437	2.290	0.988
CMK-3 10 °C	492.67	1.065	462.61	15.39	1076.1	0.933	628.83	0.416	2.402	0.999
CMK-3 20 °C	413.29	1.050	393.61	20.00	915.6	0.947	564.14	0.392	2.549	0.997
CMK-3 35 °C	396.80	1.058	374.89	17.11	872.1	0.960	585.32	0.435	2.300	0.997

**Table 3:** Sips and Toth model parameters for CO_2_ adsorption on CMK-3.

	Sips				Toth			
	*q_S_*	*K_S_*	*n_S_*	R^2^	*q_T_*	*K_T_*	*n_T_*	R^2^
	(mg·g^−1^)	(atm^−1^)			(mg·g^−1^)	(atm^−1^)		
CMK-3 0 °C	600.25	1.073	0.460	1.000	609.05	2.955	0.170	1.000
CMK-3 10 °C	612.04	1.356	0.530	1.000	616.65	3.080	0.260	1.000
CMK-3 20 °C	530.06	0.647	0.490	1.000	529.44	2.267	0.180	1.000
CMK-3 35 °C	535.32	0.661	0.530	1.000	539.43	1.940	0.180	1.000

**Table 4:** Dubinin–Radushkevich and Temkin model parameters for CO_2_ adsorption on CMK-3.

	Dubinin–Radushkevich				Temkin			
	*q_D_*	*β*	*E*	R^2^	*K_Tk_*	*B*	*b_Tk_*	R^2^
	(mg·g^−1^)	(mol^2^·kJ^−2^)	(kJ·mol^−1^)		(atm^−1^)		(kJ·mol^−1^)	
CMK-3 0 °C	601.72	0.023	4.702	0.848	53.52	162.29	0.014	0.890
CMK-3 10 °C	525.74	0.019	5.109	0.922	79.30	129.04	0.018	0.949
CMK-3 20 °C	447.22	0.016	5.679	0.958	122.52	101.04	0.024	0.978
CMK-3 35 °C	428.65	0.015	5.774	0.968	104.71	101.52	0.025	0.976

## Data Availability

The raw data supporting the conclusions of this article will be made available by the authors on request.
